# Exploratory research on drugs after lung surgery based on real-world data from the FDA adverse event reporting system database

**DOI:** 10.1371/journal.pone.0346792

**Published:** 2026-05-22

**Authors:** Qiaoling Pan, Hang Chen, Hanbo Pan, Zeyang Hu, Keyue Qiu, Jiaheng Zhang, Ni Li, Yinyu Mu, Keyun Zhu, Guodong Xu, Yiming Shen

**Affiliations:** 1 Department of Thoracic surgery, The Affiliated Lihuili Hospital of Ningbo University, Ningbo, Zhejiang Province, P. R. China; 2 Shanghai Lung Cancer Center, Shanghai Chest hospital, Shanghai Jiao Tong University School of Medicine, Shanghai, P. R. China; 3 Health Science Center, Ningbo University, Ningbo, Zhejiang Province, P. R. China; 4 Shanghai Medical College, Fudan University, Shanghai, P. R. China; Helwan University Faculty of Engineering, EGYPT

## Abstract

**Backgrounds:**

Lung surgery is the most common surgery in thoracic surgery, but postoperative dyspnea, cough, palpitations, and chest pain seriously affect the quality of life of patients and hinder their postoperative recovery.

**Methods:**

To reveal the potential correlation between drugs and adverse events, we used disproportionation analysis to calculate the ROR and PRR values of the top 30 reported cases of dyspnea, cough, palpitations, and chest pain, respectively. In addition, we defined high and medium ROR signaling drugs after lung surgery by generating Venn diagram and calculated the PRR of all drugs used to treat pulmonary hypertension to explore the correlation between pulmonary arterial hypertension drugs and these four symptoms.

**Results:**

We identified the top 30 drugs that are most related to dyspnea, cough, palpitations, and chest pain, and defined 4 high ROR signaling drugs after lung surgery, and 10 medium ROR signaling drugs after lung surgery. Among them, 3 high ROR signaling drugs are drugs for treating pulmonary hypertension (PH). Subsequently, further analysis revealed that it is the basic drug attribute of treating PH that can easily cause these four common symptoms after lung surgery.

**Conclusion:**

Our study provides a list of high and medium ROR signaling drugs drugs for postoperative patients with lung surgery and clarifies the potential correlation between the properties of drugs for treating pulmonary arterial hypertension and dyspnea, cough, palpitations, and chest pain, providing a theoretical basis for the medication of postoperative patients.

## 1. Introduction

Lung surgery has become increasingly common in recent years due to a variety of factors. Lung cancer remains the most common malignant tumor with the highest mortality rate, most of which are early-stage lung cancer, which requires surgical intervention and treatment.[1]. In addition, the rising incidence of lung diseases such as chronic obstructive pulmonary disease (COPD) [2], and emphysema has contributed to the growing demand for lung surgeries. Furthermore, advancements in medical technology and surgical techniques have also played a significant role in the increased prevalence of lung surgeries [3]. However, postoperative symptoms such as cough, chest pain, palpitations, and dyspnea are commonly observed in patients undergoing lung surgery, which can significantly damage the quality of life and recovery of patients [4]. Therefore, when administering preoperative and postoperative medications to patients undergoing pulmonary surgery, it is of paramount importance to avoid drugs with a strong correlation to the aforementioned symptoms, thereby minimizing the potential risk of exacerbating these symptoms postoperatively.

As a common complaint symptom for patients after lung surgery, dyspnea can persist for an extended period. Physically, dyspnea can limit patients’ ability to perform daily activities, leading to decreased mobility, fatigue, and reduced exercise tolerance. This respiratory distress can hinder patients’ overall functional capacity, impeding their ability to regain normal lung function and delaying their recovery. In addition to the physical limitations, dyspnea can also have a significant psychological impact on postoperative patients. The sensation of breathlessness can cause anxiety, fear, and depression, further exacerbating the distress experienced by these individuals. The psychological burden associated with dyspnea can adversely affect patients’ mental health, impede their social interactions, and decrease their overall quality of life. Appropriate and simple lung rehabilitation exercise training programs can significantly improve patients’ respiratory exercise endurance [5].

Cough is a common symptom experienced by individuals following lung surgery and can significantly impact the recovery and rehabilitation process [6]. The incidence of cough after lobectomy ranges from 18% to 50% [7]. Postoperative cough can be caused by various factors such as irritation of the airways, accumulation of mucus, or inflammation in the respiratory system [8]. While coughing is a natural protective mechanism designed to clear the airways of irritants, it can become problematic when it persists or becomes chronic [9]. Despite the prevalence and potential complications associated with postoperative cough, there is a paucity of robust clinical trials investigating its management. Current treatment options primarily focus on symptomatic relief, such as the administration of cough suppressants or expectorants [10]. Patients undergoing lung surgery are at a higher ROR signaling drugs of developing complications such as atelectasis, pneumonia, and bronchospasm. Furthermore, coughing can exacerbate these complications by placing additional strain on the respiratory system, leading to increased pain, delayed recovery, and prolonged hospital stays [11].

Palpitations, the perception of rapid or irregular heartbeats, can have a significant impact on patients’ quality of life and overall well-being, while postoperative palpitations are often caused by arrhythmia [12]. According to statistics, about 25% of patients undergoing pneumonectomy experience arrhythmia, with supraventricular tachyarrhythmia being common [13]. They are a common symptom experienced by individual’s post-pulmonary surgery, and their occurrence can be attributed to various factors such as anxiety, medication side effects, electrolyte imbalances, and cardiac arrhythmias [14]. Patients undergoing pulmonary surgery often experience a multitude of physiological changes, including alterations in heart rate and rhythm [15]. Additionally, post-operative pain and discomfort can contribute to the development of palpitations [16]. The impact of palpitations on post-pulmonary surgery patients cannot be underestimated. Palpitations can cause distress, anxiety, and fear, leading to sleep disturbances, reduced physical activity, and diminished overall quality of life [17]. Patients may experience a constant awareness of their heartbeat, leading to heightened anxiety and a persistent feeling of unease. Furthermore, palpitations can be associated with other symptoms such as dizziness, shortness of breath, and chest pain, further exacerbating the psychological and physical distress experienced by patients [18].

Chest pain is another common and distressing symptom experienced by patients after pulmonary surgery, and it can be localized to the surgical site or radiate to other areas of the chest, back, or shoulders [19,20]. According to statistics, the incidence of chronic chest pain after thoracoscopic lung surgery is about 17.4%, while the incidence of post-thoracotomy pain syndrome (PTPS) is 57% [21]. This pain can limit a patient's ability to breathe deeply, cough effectively, and engage in physical activities, thereby impacting their recovery and rehabilitation process, affecting patients’ emotional well-being, and overall quality of life, and leading to decreased mobility, impaired pulmonary function, and limited ability to perform daily activities [22]. Therefore, patients may experience anxiety, sleep disturbances, and overall reduced quality of life [23]. Additionally, the presence of chest pain can hinder the effectiveness of postoperative rehabilitation and delay recovery [24].

The FDA Adverse Event Reporting System (FAERS) is a comprehensive database that serves as a valuable resource for monitoring the safety and effectiveness of various drugs and medical products. One of the key strengths of FAERS is its ability to detect signals of potential adverse drug reactions (ADRs) that may not have been identified during pre-marketing clinical trials. The utilization of FAERS data in clinical trial research has gained significant traction in recent years. Researchers have recognized the value of integrating FAERS data with clinical trial data to gain a more comprehensive understanding of the safety profile of a drug or medical product. In the present study, we conducted an investigation to identify the top 30 drugs that are most commonly associated with cough, chest pain, palpitations, and difficulty breathing in postoperative patients by using the FAERS database.

The primary goal of this study was to determine the drugs that pose the highest risk of may exacerbating these symptoms in postoperative patients. By identifying these drugs, we can develop strategies to avoid their use and minimize the occurrence of these symptoms in lung surgery patients. Thus, we employed a Venn diagram to visualize the overlap between the drugs associated with each symptom. By examining the intersection of the drug sets, we aimed to identify the drugs that are consistently linked to multiple symptoms. This approach allowed us to avoid using the drugs that are most likely to worsen the postoperative symptoms experienced by patients. By conducting this study, we hope to provide valuable insights into the potential adverse effects of commonly used drugs in postoperative patients. Ultimately, this study aims to identify drugs strongly correlated with cough, chest pain, palpitations, and dyspnea, thereby mitigating the potential risk of exacerbating these symptoms in patients undergoing pulmonary surgery.

## 2. Materials and methods

### 2.1. Data download

This study is a retrospective study aimed at exploring drugs that may exacerbate symptoms in lung surgery patients by mining data from the FAERS database, aiming to avoid using these drugs in lung surgery patients. The FAERS data was downloaded from the FDA website (https://fis.fda.gov/extensions/FPD-QDE-FAERS/FPD-QDE-FAERS.html). The dataset used for this study includes adverse event reports from January 2018 to December 2022. The FAERS database employs a unique PRIMARYID for each case report, serving as the primary key for deduplication. We combined quarterly data files (DEMO, DRUG, REAC, etc.) by PRIMARYID to create a comprehensive dataset and Remove duplicate records based on PRIMARYID to ensure each case is represented only once in the analysis. Besides, we used the MedDRA (Medical Dictionary for Regulatory Activities) terminology set to standardize AE terms (e.g., “heart attack” → “myocardial infarction”) and assigned the appropriate MedDRA version to each term to maintain historical context and avoid discrepancies, which ensures consistency in AE reporting.

### 2.2. Data analysis

One of the key methods employed in pharmacovigilance studies is disproportionality analysis. This analytical approach aims to detect potential associations between drugs and adverse events by comparing the reporting rates of specific adverse events for a given drug with those of other drugs in the database. Reporting Odds Ratio (ROR) and the Proportional Reporting Ratio (PRR) are the key parameters used in disproportionality analysis based on FAERS database, which help to identify potential signals or associations between a drug or medical product and an adverse event. The ROR is calculated by comparing the odds of reporting a specific adverse event for a drug of interest compared to the odds of reporting the same event for all other drugs in the database. The PRR, on the other hand, compares the proportion of reports for a specific drug-adverse event combination to the proportion of reports for all other drug-event combinations in the database. A high ROR and PRR indicate a higher reporting rate for the specific drug-adverse event combination, suggesting a potential association. Pneumonectomy is the most common procedure in thoracic surgery, and patients often experience postoperative discomfort including dyspnea, cough, palpitations, and chest pain. This study calculated the ROR and PRR for the top 30 drugs associated with these four symptoms using the FAERS database (Supplementary Table 1 for calculation methods and criteria). The aim was to identify potential risk drugs that may exacerbate these symptoms post-pneumonectomy. To enhance reliability, we intersected the top 30 drugs for each symptom and generated a Venn diagram to classify high and medium ROR signaling drugs post-surgery. Drugs ranking in the top 30 for all four symptoms were defined as high ROR signaling drugs, while those in the top 30 for three symptoms were defined as medium ROR signaling drugs. This provides thoracic surgeons with theoretical evidence and significant clinical guidance for drug selection.

## 3. Results

We have identified the top 30 most common drugs that cause dyspnea, coughing, palpitations, and chest pain, and we define the top 30 drugs that are most likely to cause a certain symptom as high ROR signaling drugs for that symptom. The high ROR signaling drugs for dyspnea included Spiriva (ROR = 8.87 (7.92-9.93)), Salbutamol (ROR = 7.65 (7.41-7.91)), Entresto (ROR = 3.73 (3.62-3.83)), Opsumit (ROR = 4.41 (4.28-4.55)), Xolair (ROR = 2.52 (2.42-2.62)), Ambrisentan (ROR = 6.08 (5.86-6.31)), Remodulin(ROR = 5.04 (4.82-5.27)), Symbicort (ROR = 6.48 (6.20-6.78)), Tyvaso (ROR = 6.25(5.95-6.56)), Ibrance (ROR = 1.16 (1.11-1.22)), Uptravi (ROR = 3.69 (3.51-3.88)), Ofev (ROR = 3.03 (2.88-3.19)), Trelegy Ellipta (ROR = 6.48 (6.20-6.78)), Breo Ellipta (ROR = 7.02 (6.62-7.44)), Advair Diskus (ROR = 6.69 (6.35-7.04)), Pomalyst (ROR = 1.59 (1.50-1.69)), Nucala (ROR = 2.31 (1.92-2.78)), Anoro Ellipta (ROR = 7.39 (6.91-7.91)), Oxaliplatin (ROR = 3.45 (2.64-4.50)), Brilinta (ROR = 7.00 (6.58-7.44)), Carboplatin (ROR = 2.32 (2.18-2.46)), Copaxone (ROR = 2.57 (2.41-2.74)), Zejula (ROR = 1.18 (1.10-1.26)), Ibuprofen (ROR = 1.30 (1.22-1.39)), Veletri (ROR = 3.20 (3.01-3.41)), Orenitram (ROR = 1.29 (1.19-1.35)), Nivolumab (ROR = 1.19 (1.12-1.26)), Arikayce (ROR = 5.04 (4.72-5.39)), Paclitaxel (ROR = 3.46 (3.27-3.66)), and Adempas (ROR = 3.50 (3.24-3.78)) (Table 1).

**Table 1:** Top 30 medications associated with dyspnea from the FAERS arranged by frequency, Jan, 2018 to Dec, 2022.

Ranking	Medication	Frequency	ROR(95% two-sided CI)	PRR
1	Spiriva	1465	8.87(7.92-9.93)	8.80
2	Salbutamol	1419	7.65(7.41-7.91)	7.24
3	Entresto	5797	3.73(3.62-3.83)	3.64
4	Opsumit	4134	4.41(4.28-4.55)	4.29
5	Xolair	2458	2.52(2.42-2.62)	2.49
6	Ambrisentan	2305	6.08(5.86-6.31)	5.82
7	Remodulin	2081	5.04(4.82-5.27)	4.87
8	Symbicort	2049	6.48(6.20-6.78)	6.19
9	Tyvaso	1769	6.25(5.95-6.56)	5.98
10	Ibrance	1565	1.16(1.11-1.22)	1.16
11	Uptravi	1460	3.69(3.51-3.88)	3.61
12	Ofev	1445	3.03(2.88-3.19)	2.98
13	Trelegy Ellipta	1407	6.48(6.20-6.78)	6.19
14	Breo Ellipta	1218	7.02(6.62-7.44)	6.67
15	Advair Diskus	1183	6.69(6.35-7.04)	6.37
16	Pomalyst	1097	1.59(1.50-1.69)	1.58
17	Nucala	997	2.31(1.92-2.78)	2.31
18	Anoro Ellipta	892	7.39(6.91-7.91)	7.00
19	Oxaliplatin	887	3.45(2.64-4.50)	3.38
20	Brilinta	880	7.00(6.58-7.44)	6.65
21	Carboplatin	853	2.32(2.18-2.46)	2.30
22	Copaxone	846	2.57(2.41-2.74)	2.53
23	Zejula	821	1.18(1.10-1.26)	1.17
24	Ibuprofen	817	1.30(1.22-1.39)	1.30
25	Veletri	804	3.20(3.01-3.41)	3.14
26	Orenitram	781	1.29(1.19-1.35)	1.29
27	Nivolumab	757	1.19(1.12-1.26)	1.19
28	Arikayce	712	5.04(4.72-5.39)	4.87
29	Paclitaxel	707	3.46(3.27-3.66)	3.39
30	Adempas	688	3.50(3.24-3.78)	3.43

The high ROR signaling drugs for cough included Entresto (ROR = 5.49 (5.32-5.67)), Cosentyx (ROR = 1.69 (1.62-1.76)), Dupixent (ROR = 1.38 (1.33-1.44)), Xolair (ROR = 3.51 (3.35-3.67)), Revlimid (ROR = 1.09 (1.04-1.15)), Tyvaso (ROR = 10.19 (9.68-10.73)), Repatha (ROR = 1.55 (1.46-1.63)), Opsumit (ROR = 2.37 (2.24-2.51)), Paxlovid (ROR = 3.75 (3.55-4.01)), Ibrance (ROR = 1.62(1.53-1.71)), Xeljanz (ROR = 1.65 (1.59-1.73)), Ocrevus (ROR = 1.99 (1.88-2.12)), Ofev (ROR = 4.02 (3.78-4.27)), Gilenya (ROR = 1.39 (1.30-1.48)), Esbriet (ROR = 1.35 (1.29-1.41)), Inflectra (ROR = 1.28 (1.22-1.34)), Abatacept (ROR = 3.72 (3.52-3.94)), Breo Ellipta (ROR = 5.97 (5.50-6.49)), Nucala (ROR = 4.52 (4.26-4.80)), Rituximab (ROR = 1.23 (1.16-1.29)), Salbutamol (ROR = 4.19 (3.96-4.44)), Trikafta (ROR = 5.10 (4.63-5.61)), Advair Diskus (ROR = 4.51 (4.16-4.90)), Zejula (ROR = 1.11 (1.01-1.23)), Sandostatin Lar Depot (ROR = 1.41 (1.29-1.53)), Anoro Ellipta (ROR = 6.14 (5.56-6.77)), Ambrisentan (ROR = 1.89 (1.73-2.06)), Rinvoq (ROR = 1.22 (1.10-1.35)), Kisqali (ROR = 1.76 (1.60-1.95)), and Alemtuzumab (ROR = 1.50 (1.35-1.68)) (Table 2).

**Table 2:** Top 30 medications associated with cough from the FAERS arranged by frequency, Jan, 2018 to Dec, 2022.

Ranking	Medication	Frequency	ROR(95% two-sided CI)	PRR
1	Entresto	5351	5.49(5.32-5.67)	5.38
2	Cosentyx	2325	1.69(1.62-1.76)	1.69
3	Dupixent	1994	1.38(1.33-1.44)	1.38
4	Xolair	1809	3.51(3.35-3.67)	3.47
5	Revlimid	1612	1.09(1.04-1.15)	1.09
6	Tyvaso	1537	10.19(9.68-10.73)	9.77
7	Repatha	1271	1.55(1.46-1.63)	1.54
8	Opsumit	1244	2.37(2.24-2.51)	2.36
9	Paxlovid	1209	3.75(3.55-4.01)	3.62
10	Ibrance	1157	1.62(1.53-1.71)	1.61
11	Xeljanz	1055	1.65(1.59-1.73)	1.65
12	Ocrevus	1037	1.99(1.88-2.12)	1.98
13	Ofev	1037	4.02(3.78-4.27)	3.96
14	Gilenya	867	1.39(1.30-1.48)	1.39
15	Esbriet	804	1.35(1.29-1.41)	1.35
16	Inflectra	738	1.28(1.22-1.34)	1.27
17	Abatacept	572	3.72(3.52-3.94)	3.67
18	Breo Ellipta	570	5.97(5.50-6.49)	5.84
19	Nucala	535	4.52(4.26-4.80)	4.45
20	Rituximab	534	1.23(1.16-1.29)	1.23
21	Salbutamol	479	4.19(3.96-4.44)	4.13
22	Trikafta	424	5.10(4.63-5.61)	5
23	Advair Diskus	421	4.51(4.16-4.90)	4.44
24	Zejula	416	1.11(1.01-1.23)	1.11
25	Sandostatin Lar Depot	415	1.41(1.29-1.53)	1.4
26	Anoro Ellipta	408	6.14(5.56-6.77)	5.99
27	Ambrisentan	388	1.89(1.73-2.06)	1.88
28	Rinvoq	380	1.22(1.10-1.35)	1.22
29	Kisqali	359	1.76(1.60-1.95)	1.76
30	Alemtuzumab	243	1.50(1.35-1.68)	1.5

The high ROR signaling drugs for palpitation included Tymlos (ROR = 16.66 (15.56-17.83)), Entresto (ROR = 1.50 (1.36-1.65)), Opsumit (ROR = 2.62 (2.40-2.86)), Zejula (ROR = 2.92 (2.64-3.23)), Gilenya (ROR = 1.65 (1.49-1.83)), Ciprofloxacin (ROR = 3.65 (3.30-4.03)), Copaxone (ROR = 4.18 (3.72-4.70)), Veletri (ROR = 4.87 (4.33-5.47)), Amlodipine (ROR = 3.11 (2.82-3.42)), Synthroid (ROR = 2.15 (1.81-2.57)), Ambrisentan (ROR = 3.06 (2.73-3.44)), Xyrem (ROR = 1.68 (1.47-1.92)), Remodulin (ROR = 2.68 (2.35-3.06)), Ocrevus (ROR = 1.17 (1.03-1.34)), Sertraline (ROR = 3.39 (3.04-3.77)), Imbruvica (ROR = 1.22 (1.07-1.40)), Orenitram (ROR = 3.25 (2.99-3.53)), Uptravi (ROR = 2.33 (2.02-2.68)), Ibuprofen (ROR = 1.51 (1.32-1.73)), Tyvaso (ROR = 2.64 (2.24-3.10)), Metoprolol (ROR = 6.42 (5.75-7.16)), Levofloxacin (ROR = 2.99 (2.60-3.45)), Clarithromycin (ROR = 4.29 (3.63-5.07)), Emgality (ROR = 1.90 (1.73-2.09)), Bisoprolol (ROR = 4.55 (3.95-5.25)), Metoprolol Succinate (ROR = 8.56 (7.33-10.01)), Northera (ROR = 2.58 (2.10-3.17)), Ramipril (ROR = 2.12 (1.75-2.56)), Ingrezza (ROR = 1.42 (1.15-1.75)), and Tasigna (ROR = 1.39 (1.13-1.71)) (Table 3).

**Table 3:** Top 30 medications associated with palpitations from the FAERS arranged by frequency, Jan, 2018 to Dec, 2022.

Ranking	Medication	Frequency	ROR(95% two-sided CI)	PRR
1	Tymlos	870	16.66(15.56-17.83)	16.25
2	Entresto	486	1.50(1.36-1.65)	1.5
3	Opsumit	478	2.62(2.40-2.86)	2.61
4	Zejula	382	2.92(2.64-3.23)	2.91
5	Gilenya	366	1.65(1.49-1.83)	1.65
6	Ciprofloxacin	302	3.65(3.30-4.03)	3.63
7	Copaxone	266	4.18(3.72-4.70)	4.16
8	Veletri	257	4.87(4.33-5.47)	4.84
9	Amlodipine	256	3.11(2.82-3.42)	3.10
10	Synthroid	227	2.15(1.81-2.57)	2.15
11	Ambrisentan	223	3.06(2.73-3.44)	3.05
12	Xyrem	220	1.68(1.47-1.92)	1.68
13	Remodulin	217	2.68(2.35-3.06)	2.67
14	Ocrevus	215	1.17(1.03-1.34)	1.17
15	Sertraline	211	3.39(3.04-3.77)	3.37
16	Imbruvica	188	1.22(1.07-1.40)	1.22
17	Orenitram	186	3.25(2.99-3.53)	3.24
18	Uptravi	181	2.33(2.02-2.68)	2.32
19	Ibuprofen	162	1.51(1.32-1.73)	1.51
20	Tyvaso	148	2.64(2.24-3.10)	2.63
21	Metoprolol	128	6.42(5.75-7.16)	6.36
22	Levofloxacin	126	2.99(2.60-3.45)	2.98
23	Clarithromycin	123	4.29(3.63-5.07)	4.27
24	Emgality	120	1.90(1.73-2.09)	1.90
25	Bisoprolol	108	4.55(3.95-5.25)	4.52
26	Metoprolol Succinate	102	8.56(7.33-10.01)	8.46
27	Northera	90	2.58(2.10-3.17)	2.57
28	Ramipril	90	2.12(1.75-2.56)	2.11
29	Ingrezza	86	1.42(1.15-1.75)	1.42
30	Tasigna	86	1.39(1.13-1.71)	1.39

The high ROR signaling drugs for chest pain included Entresto (ROR = 2.74 (2.58-2.91)), Opsumit (ROR = 3.26 (3.05-3.48)), Repatha (ROR = 1.27 (1.17-1.38)), Ambrisentan (ROR = 4.40 (4.06-4.77)), Gilenya (ROR = 1.41 (1.29-1.55)), Xolair (ROR = 1.55 (1.41-1.70)), Remodulin (ROR = 3.45 (3.13-3.81)), Tyvaso (ROR = 4.59 (4.14-5.09)), Uptravi (ROR = 2.71 (2.43-3.02)), Ofev (ROR = 2.17 (1.94-2.43)), Veletri (ROR = 3.68 (3.29-4.11)), Brilinta (ROR = 7.33 (6.56-8.19)), Rinvoq (ROR = 1.61 (1.42-1.82)), Sandostatin Lar Depot (ROR = 1.44 (1.28-1.62)), Copaxone (ROR = 2.57 (2.27-2.90)), Ibuprofen (ROR = 1.32 (1.17-1.49)), Tasigna (ROR = 2.40 (2.11-2.73)), Pomalyst (ROR = 1.08 (0.94-1.24)), Orenitram (ROR = 1.32 (1.17-1.49)), Ranolazine (ROR = 14.64 (12.84-16.69)), Oxbryta (ROR = 1.92 (1.66-2.23)), Adempas (ROR = 3.27 (2.83-3.79)), Ramipril (ROR = 2.86 (2.50-3.27)), Benlysta (ROR = 1.93 (1.67-2.23)), Oxaliplatin (ROR = 1.81 (1.57-2.08)), Paclitaxel (ROR = 2.36 (2.07-2.68)), Tymlos (ROR = 1.78 (1.51-2.10)), Symbicort (ROR = 1.50 (1.26-1.77)), Ciprofloxacin (ROR = 1.27 (1.10-1.46)), and Trelegy Ellipta (ROR = 1.50 (1.26-1.77)) (Table 4).

**Table 4:** Top 30 medications associated with chest pain from the FAERS arranged by frequency, Jan, 2018 to Dec, 2022.

Ranking	Medication	Frequency	ROR(95% two-sided CI)	PRR
1	Entresto	1189	2.74(2.58-2.91)	2.73
2	Opsumit	854	3.26(3.05-3.48)	3.24
3	Repatha	538	1.27(1.17-1.38)	1.27
4	Ambrisentan	495	4.40(4.06-4.77)	4.36
5	Gilenya	455	1.41(1.29-1.55)	1.41
6	Xolair	425	1.55(1.41-1.70)	1.54
7	Remodulin	404	3.45(3.13-3.81)	3.43
8	Tyvaso	371	4.59(4.14-5.09)	4.55
9	Uptravi	306	2.71(2.43-3.02)	2.69
10	Ofev	290	2.17(1.94-2.43)	2.17
11	Veletri	273	3.68(3.29-4.11)	3.65
12	Brilinta	268	7.33(6.56-8.19)	7.22
13	Rinvoq	257	1.61(1.42-1.82)	1.61
14	Sandostatin Lar Depot	237	1.44(1.28-1.62)	1.44
15	Copaxone	230	2.57(2.27-2.90)	2.56
16	Ibuprofen	217	1.32(1.17-1.49)	1.32
17	Tasigna	208	2.40(2.11-2.73)	2.39
18	Pomalyst	203	1.081069977	1.29
19	Orenitram	200	1.32(1.17-1.49)	1.32
20	Ranolazine	198	14.64(12.84-16.69)	14.17
21	Oxbryta	179	1.92(1.66-2.23)	1.92
22	Adempas	178	3.27(2.83-3.79)	3.26
23	Ramipril	178	2.86(2.50-3.27)	2.84
24	Benlysta	172	1.93(1.67-2.23)	1.93
25	Oxaliplatin	156	1.81(1.57-2.08)	1.80
26	Paclitaxel	144	2.36(2.07-2.68)	2.35
27	Tymlos	141	1.78(1.51-2.10)	1.77
28	Symbicort	137	1.50(1.26-1.77)	1.49
29	Ciprofloxacin	136	1.27(1.10-1.46)	1.27
30	Trelegy Ellipta	135	1.50(1.26-1.77)	1.49

**Fig 1:**
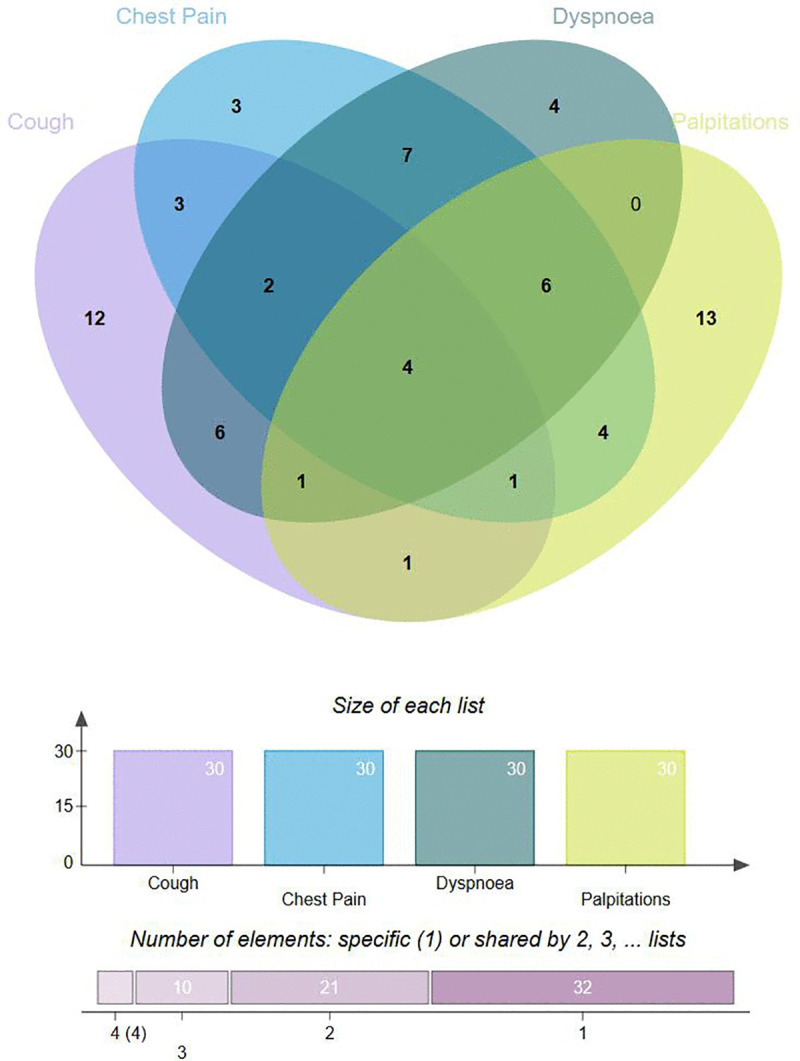
Venn diagram of the top 30 medications that intersect dyspnea, cough, palpitations, and chest pain.

According to the Venn diagram in Fig 1, after intersecting the four symptoms, we found that Ambrisentan, Opsumit, Tyvaso, and Entresto were all high ROR signaling drugs for these four symptoms. We defined these four drugs as high ROR signaling drugs after lung surgery, among which the first three are drugs for treating pulmonary arterial hypertension, while Entresto is a commonly used drug for treating chronic heart failure in clinical practice. In addition, we refer to drugs that meet all three symptoms as medium ROR signaling drugs after lung surgery, and found that Orenitram, Ibuprofen, Copaxone, Uptravi, Remodulin, and Veletri were high ROR signaling drugs for chest pain, dyspnea, and palpitations; Zejula is a high ROR signaling drug for cough, dyspnoea, and palpitations; Gilenya was a high ROR signaling drug for cough, chest pain, and palpitations; and Xoair and Ofev were high ROR signaling drugs for cough, chest pain, and dyspnoea.

**Fig 2:**
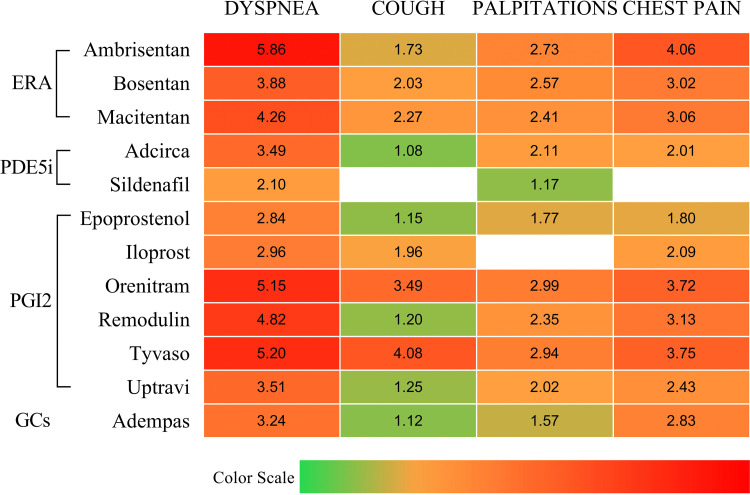
Heatmap of the RORL values of all drugs used to treat PH in dyspnea, cough, palpitations, and chest pain.

Interestingly, out of the four high ROR signaling drugs, three are drugs for treating pulmonary hypertension (PH). To investigate whether this situation occurs by chance or is related to the basic properties of drugs for treating PH, we calculated the ROR values of all anti PH drugs for the postoperative symptoms of these four types of lung surgery, respectively. We found that out of the 12 drugs currently approved by the FDA for the treatment of PH (Adcirca, Adempas, Ambrisentan, Bosentan, Epoprostenol, Iloprost, Macitentan, Orenitram, Remodulin, Sildenafil, Tyvaso, Uptravi), 9 drugs are closely related to dyspnea, cough, palpitations, and chest pain (Table 5). Among the remaining three drugs, Iloprost is closely related to dyspnea, cough, and chest pain, Riociguat is closely related to cough, palpitations, and chest pain, while Sildenafil is only related to dyspnea and palpitations. From this, we speculate that it is the properties of PH drugs that are closely related to dyspnea, coughing, palpitations, and chest pain (Fig 2).

**Table 5:** The ROR values of 12 drugs used to treat PH in dyspnea, cough, palliations, and chest pain.

ROR	DYSPNEA	COUGH	PALPITATIONS	CHEST PAIN
Ambrisentan	6.08(5.86-6.31)	1.89(1.73-2.06)	3.06(2.73-3.44)	4.40(4.06-4.77)
Bosentan	4.11(3.88-4.35)	2.24(2.03-2.49)	2.98(2.57-3.46)	3.40(3.02-3.81)
Macitentan	4.40(4.26-4.54)	2.40(2.27-2.54)	2.64(2.41-2.89)	3.27(3.06-3.50)
Adcirca	3.76(3.49-4.05)	1.29(1.08-1.52)	2.58(2.11-3.16)	2.40(2.01-2.85)
Sildenafil	2.28(2.10-2.48)	0.88(0.73-1.05)	1.47(1.17-1.85)	1.16(0.94-1.44)
Epoprostenol	3.36(2.84-3.99)	1.60(1.15-2.24)	2.72(1.77-4.18)	2.59(1.80-3.73)
Iloprost	3.49(2.96-4.10)	2.53(1.96-3.27)	1.32(0.73-2.39)	2.91(2.09-4.06)
Orenitram	5.30(5.15-5.46)	3.66(3.49-3.84)	3.25(2.99-3.53)	3.96(3.72-4.21)
Remodulin	5.04(4.82-5.27)	1.35(1.20-1.51)	2.68(2.35-3.06)	3.45(3.13-3.81)
Tyvaso	5.35(5.20-5.51)	4.26(4.08-4.44)	3.19(2.94-3.47)	3.99(3.75-4.24)
Uptravi	3.69(3.51-3.88)	1.39(1.25-1.55)	2.33(2.02-2.68)	2.71(2.43-3.02)
Adempas	3.50(3.24-3.78)	1.32(1.12-1.56)	1.97(1.57-2.48)	3.27(2.83-3.79)

## 4. Discussion

Lung surgery is a common procedure performed to treat various lung conditions to improve the patient's overall health and quality of life, but it is not without potential complications and adverse effects [25]. One of the primary concerns following lung surgery is the occurrence of common symptoms that can significantly impact the patient's recovery and well-being. These symptoms can range from mild discomfort to severe complications, leading to prolonged hospital stays and increased healthcare costs [26]. In our study, we defined dyspnea, cough, palpations, and chest pain as common postoperative symptoms of lung surgery and aimed to reduce the risk of common postoperative symptoms and improve patients’ quality of life by exploring the drugs that are most related to these four symptoms.

In this study, we analyzed the data from the FAERS database through disproportionation analysis and identified the top 30 drugs most commonly realted to dyspnea, cough, palpations, and chest pain, respectively, which were defined as high ROR signaling drugs with corresponding symptoms. Subsequently, by generating a Venn diagram, we defined drugs that were simultaneously on the high ROR signaling drugs list of four symptoms as high ROR signaling drugs after lung surgery, while drugs that met the high ROR signaling drugs list of three symptoms were defined as medium ROR signaling drugs after lung surgery. We have identified 4 high ROR signaling drugs after lung surgery and 10 medium ROR signaling drugs after lung surgery, among which 3 high ROR signaling drugs are used to treat PH. At present, there has been no systematic discussion on the correlation between drugs for treating pulmonary arteries and postoperative symptoms of lung surgery. In order to further explore whether the basic properties of PH drugs are closely related to postoperative symptoms of lung surgery, we explored the ROR values of 12 drugs currently available for the treatment of PH and dyspnea, cough, medications, and chest pain, and found that 10 drugs are closely related to all four symptoms. Therefore, we speculate that the basic properties of PH drugs may have a potential correlation with postoperative symptoms of lung surgery.

PH is a chronic and progressive disease characterized by elevated pressure in the pulmonary arteries, leading to right heart dysfunction and ultimately, heart failure, with an estimated prevalence of 15–50 cases per million individuals worldwide [27]. Although the exact etiology of PH remains unclear, genetic mutations and hereditary factors are known to play a significant role in the development of PH [28]. Additionally, other risk factors for PH include chronic lung diseases (e.g., COPD), connective tissue disorders (e.g., systemic sclerosis), human immunodeficiency virus (HIV) infection, and exposure to certain drugs or toxins [29–31]. Clinical manifestations of PH can vary depending on the underlying cause and the stage of the disease. Common symptoms of PH include dyspnea, fatigue, chest pain, palpitations, and syncope, and patients may experience worsening symptoms, exercise intolerance, and right-sided heart failure in the development of PH [32]. The drug treatment for PH mainly includes vasodilators and specific receptor agonists. Vasodilators include endothelin receptor antagonists (ERA), phosphodiesterase type 5 inhibitors (PDE5i), and prostanoids and prostacyclins (PGI2). Specific receptor agonists include Guanylate cyclase (GCs) agonists [33].

ERAs are a class of drugs that can antagonize the cell proliferation and pulmonary vasoconstriction effects of endothelin, including Ambrisentan, Bosentan, Macitentan, which have played a significant role in the treatment of pulmonary arterial hypertension [34]. Studies have suggested that endothelin receptor antagonists may block diuretic effects through endothelin A and B receptors in the renal collection tube, leading to peripheral edema, such as pulmonary edema, which can lead to dyspnea and cough in patients [35,36]. In addition, on the one hand, they can cause anemia, but the specific mechanism is unclear [37]. On the other hand, it can cause systemic vascular dilation, which may cause a decrease in blood pressure [38]. Both mechanisms may cause patients to experience palpitations. In addition, in patients with pulmonary edema, excessive fluid accumulation in the lungs can lead to excessive pressure in the chest cavity, which can pull on surrounding tissues and ligaments, leading to chest pain [39].

PDE5i, represented by Adcirca, Sildenafil, can inhibit phosphodiesterase type 5 (PDE5), enhance the effect of nitric oxide (NO), relax vascular smooth muscle, expand pulmonary blood vessels selectively, and reduce pulmonary circulation resistance in patients with PH [40]. Although PDE5i can suppress PDE5 in lung tissue, induce pulmonary artery dilation, and alleviate pulmonary artery pressure, PDE5i can cause changes in lung function, leading to complications such as dyspnea and cough [41]. However, PDE5i may suppress PDE1 in myocardial cells and PDE5 in vascular smooth muscle by crossing, reducing the degradation of cGMP and cAMP in the body, leading to increased heart rate, vascular dilation, and ultimately leading to symptoms of palpitations in patients [42]. In addition, a Chinese news report reported that patients experienced symptoms of chest pain after high dose use of PDE5i. The reason is that PDE5i causes redistribution of arterial blood flow while expanding the pulmonary artery, leading to insufficient coronary artery perfusion and triggering chest pain [43].

PGI2 is an effective vasodilator with broad application prospects in the treatment of PH, including Epoprostenol, Iloprost, Oreniram, Remodelin, Tyvaso, and Uptravi [44]. However, PGI2 may induce contraction of tracheal smooth muscle during the onset of action, causing irritating cough, and in severe cases, may lead to dyspnea [45]. In addition, PGI2 drugs can inhibit the growth of vascular smooth muscle cells and platelet aggregation, causing an increase in cyclic adenosine monophosphate (cAMP) in vascular smooth muscle cells, which relaxes pulmonary and systemic arterial blood vessels, leading to hypotension [46]. Therefore, some patients may experience symptoms of palpitations when receiving PGI2 treatment. PGI2 drugs can also cause local vascular dilation and muscle contraction in the chest, causing chest pain and discomfort, and some female patients may experience breast pain and nipple pain [47].

GCs agonists, represented by Adempas, can directly stimulate guanylate cyclase and enhance its sensitivity to low levels of NO, and NO may cause airway hyperresponsiveness and bronchial asthma in patients, leading to dyspnea and cough [48,49]. Like other drugs used to treat PH, it is generally believed that the occurrence of palpitations in patients undergoing GCs agonists treatment may be due to the dilation effect of GCs agonists on the patient's systemic arteries, causing hypotension and dizziness [50]. Additionally, during the treatment with GCs antibiotics, patients may experience excessive dilation of the pulmonary arteries, leading to symptoms of chest pain during treatment [51].

As is well known, lung cancer has become the malignancy with the highest incidence and mortality globally. However, minimally invasive surgical resection remains the gold standard for treating early-stage and locally advanced NSCLC. Nevertheless, most patients experience postoperative discomforts such as dyspnea, cough, palpitations, and chest pain. On one hand, it is crucial to administer symptomatic medications to alleviate these symptoms; on the other hand, it is equally important to avoid medications that may exacerbate these symptoms. Based on the fundamental characteristics of PAH medications and the calculation of the ROR for these symptoms in this study, we speculate that the use of PAH medications in patients undergoing lung resection surgery may potentially worsen postoperative dyspnea, cough, palpitations, and chest pain. What’s more, it is worth noting that sildenafil is only associated with dyspnea and palpitations, and it is the drugs used to treat PH causing the least postoperative symptoms and having the lowest ROR value among all drugs.

Our study is the first to investigate the top 30 drugs associated with dyspnea, cough, palpitations, and chest pain, respectively. Notably, this study calculates the ROR and PRR values through disproportionality analysis, which effectively identifies potential signals or associations between drugs and adverse events. The drugs with high and medium ROR signals explored in this study aim to investigate the correlation between target drugs and adverse events, rather than causal relationships. Therefore, the results of this study cannot guide clinical medication use.

However, our research also has several limitations. Firstly, the data in the FAERS database is voluntary in nature, and this voluntary reporting system may introduce reporting bias, resulting in insufficient representation of adverse events in the database. Therefore, the results of this study are inevitably subject to data bias. The data findings are suggestive, but there remains a long way to go before they can guide clinicians in medication management. Multi-database platform joint analysis will also be the next research direction for our team. Secondly, the results obtained are limited to the analysis of online databases, without real world data to verify, such as the specific mechanisms and signaling pathways of dyspnea, cough, palpitations, and chest pain, which is exactly the future research direction of our team. Finally, the reported adverse drug reactions (ADRs) in the FAERS database are often one-sided, as there is a significant correlation between ADRs and patients’ health conditions, duration of medication, and drug dosage. Therefore, it is essential to validate the results from the FAERS database with real-world outcomes.

In summary, our study identified 4 high ROR signaling drugs after lung surgery and 10 medium ROR signaling drugs after lung surgery. In addition, our research revealed that the basic properties of drugs used to treat PH may exacerbate common symptoms after lung surgery, which provided a theoretical basis for clinical clinicians to prescribe medication for patients after lung surgery.

## Supporting information

S1 Table Two major algorithms used for signal detection. (DOCX)

## Abbreviations

COPD: chronic obstructive pulmonary disease
PTPS: post-thoracotomy pain syndrome
FAERS: FDA Adverse Event Reporting System
ADRs: adverse drug reactions
ROR: Reporting Odds Ratio
PRR: Proportional Reporting Ratio
PH: pulmonary hypertension
HIV: human immunodeficiency virus
ERA: endothelin receptor antagonists
PDE5i: phosphodiesterase type 5 inhibitors
GCs: Guanylate cyclase
PDE5: phosphodiesterase type 5
NO: nitric oxide
cAMP: cyclic adenosine monophosphate
