# Supplementary material for: Exploratory research on drugs after lung surgery based on real-world data from the FDA adverse event reporting system database
Source: PLoS One. 2026 May 22;21(5):e0346792. doi: 10.1371/journal.pone.0346792 (PMC13196928; doi:10.1371/journal.pone.0346792)
Supplement: S1 Table — (DOCX) [file pone.0346792.s001.docx]

**Supplementary Table 1. Two major algorithms used for signal detection.**

| Algorithms | Equation | Criteria |
| --- | --- | --- |
| ROR | ROR=ad/bc | lower limit of 95% CI>1, N≥10 |
|  | 95%CI=e^ln(ROR)±1.96(1/a+1/b+1/c+1/d)^0.5^ |  |
| PRR | PRR=a(c+d)/c/(a+b) | PRR≥2, χ^2^≥4, N≥10 |
|  | χ^2^=[(ad-bc)^2](a+b+c+d)/[(a+b)(c+d)(a+c)(b+d)] |  |

Equation: a, number of reports containing both the target drug and target adverse drug reaction; b, number of reports containing other adverse drug reaction of the target drug; c, number of reports containing the target adverse drug reaction of other drugs; d, number of reports containing other drugs and other adverse drug reactions. 95%CI, 95% confidence interval; N, the number of reports; χ^2^, chi-squared.
